# Genome-wide association study of salt tolerance in sorghum during germination

**DOI:** 10.3389/fpls.2025.1682270

**Published:** 2025-12-10

**Authors:** Lihua Wang, Zhichao Xing, Jiajie Zhou, Min Jiang, Qiwu Fan, Guobao Yang, Long Li, Yunlong Wang, Ephrem Habyarimana, Yongfei Wang, Die Hu, Yi-Hong Wang

**Affiliations:** 1 College of Agriculture, Anhui Science and Technology University, Chuzhou, Anhui, China; 2 International Joint Research Center of Forage Bio-Breeding in Anhui Province, Chuzhou, China; 3 The International Crops Research Institute for the Semi-Arid Tropics (ICRISAT), Patancheru, Telangana, India; 4 Department of Biology, University of Louisiana at Lafayette, Lafayette, LA, United States

**Keywords:** sorghum, mini core, GWAS, salt tolerance, seedling

## Abstract

**Introduction:**

Salt stress is a major abiotic factor restricting sorghum seed germination and early seedling establishment, particularly in saline-affected soils. Understanding the genetic architecture underlying salt tolerance during germination is essential for improving sorghum adaptation to saline environments. Genome-wide association studies (GWAS) based on high-density genomic variants provide an effective approach for uncovering loci and genes controlling complex stress-response traits. However, the genetic basis of sorghum salt tolerance at the seedling stage remains insufficiently characterized.

**Methods:**

To dissect the genetic architecture of salt tolerance during germination, we conducted a genome-wide association study (GWAS) using a panel of 245 sorghum mini core accessions and 6,094,317 high-quality SNPs obtained through whole genome resequencing. Seedlings were evaluated under five NaCl concentrations (0, 50, 100, 150, and 200 mmol/L) in 2019 and three (0, 50, and 200 mmol/L) in 2020 for shoot/root length, shoot/root fresh weight, and shoot/root dry weight, resulting in 84 trait/treatment/year combinations for GWAS.

**Results and Discussion:**

GWAS mapped 35 salt tolerance loci and 39 candidate genes were identified for salt tolerance from 29 of the 35 loci. Majority of these candidate genes (29 of the 39) have orthologs in other species that have been shown to play roles in salt tolerance in plants. These candidate genes potentially involved in ion transport, stress signaling, and growth regulation were identified in genomic regions in or adjacent to the location of associated markers. These findings provide valuable insights into the genetic basis of salt tolerance in sorghum and offer potential targets for marker-assisted selection and genetic improvement of salt-tolerant cultivars.

## Introduction

Crop yield can be significantly reduced by soil salinity which can occur naturally from the retention of soluble salt in the soil (Amombo et al., 2022) and also as a result of irrigation practices due to increased evaporation in periods of drought (van Zelm et al., 2020). Globally, abiotic stresses including soil salinity can cause as much as 80% yield reduction in sorghum (Zurbriggen et al., 2010). Salt stress negatively affects sorghum growth and yield. It can reduce germination rate by 80% depending on salt concentration (Chen et al., 2022). Yield reduction is also positively correlated with degree of salinity. For example, biomass yield in sorghum can be reduced by 11-33% at 60 mM NaCl but 30-58% at 120 mM NaCl (Rajabi Dehnavi et al., 2024). Similarly, sorghum grain yield can be reduced by half when grown in saline soils (Daniells et al., 2001). Because of salt’s negative impact on sorghum growth, shoot growth (Wang et al., 2025) at germination phase (Mansour et al., 2021) has been demonstrated to be reliable measure of salt tolerance in sorghum although the molecular mechanisms of how salinity affect shoot growth is still poorly understood (van Zelm et al., 2020; Yu et al., 2023).

Plant cellular response to salinity can be broken down into four phases. Early salt sensing is within 5 min of salt stress application. Then from 5 min to 5 hours (h) after salt exposure, a period called stop phase, root growth rate decreases, stays decreased in the quiescent phase 5–9 h after salt exposure, and partly recovers during the ensuing growth recovery phase 9 h after salt exposure (van Zelm et al., 2020). The immediate effect of salt stress is three-fold: it produces osmotic stress, causes cytotoxic accumulation of reactive oxygen species (ROS), as well as Na^+^/Cl^-^ toxicity (Amombo et al., 2022; van Zelm et al., 2020). Plants confront ROS by activating antioxidant genes, osmotic stress by genetic pathways as well as changes in root system architecture, and Na^+^/Cl^-^ toxicity by Na^+^ sequestration in the vacuoles through Na^+^/H^+^ antiporter (Amombo et al., 2022; Mansour et al., 2021; Ukwatta et al., 2021; van Zelm et al., 2020). ROS may cause double-stranded break in DNA as a result of salt stress (Zvanarou et al., 2020).

Among the grasses, mapping of salt tolerance has been extensively performed in rice. Kong et al. (2021) reported a total of seven QTLs (quantitative trait loci) identified on chromosome 3, 4, 5, 6, and 8 while Yu et al. (2018) revealed 12 such loci. Other studies reported far more salt tolerance QTLs. Yuan et al. (2020) identified 21 QTLs, and Cui et al. (2018) and Lv et al. (2022) found 56 and 155 significant SNPs, respectively. Two groups have conducted salt tolerance mapping in sorghum. Wang et al. (2014, 2020) mapped six major QTLs for salt tolerance in sorghum using 181 recombinant inbred lines. Similarly, Hostetler et al. (2021) identified 10 salt tolerance QTLs in sorghum. Searching the Sorghum QTL Atlas (Mace et al., 2019) only turned up one salt tolerance QTL identified by Wang et al. (2014) and listed only one locus on chromosome 7 (7:59547957-61793814) not overlapping with loci mapped in this study. This suggests that sorghum salt tolerance research is lagging behind other crops.

Candidate genes were also being identified. Using bulked segregation analysis (BSA) sequencing, Zhang et al. (2022) pinpointed Sobic.001G283700 encoding glycerol-3-phosphate acyltransferase as the salt tolerance gene in sorghum. By comparing two sorghum genotypes, Ren et al. (2022) suggested that flavonoid pathways may play an important physiological role in sorghum salt tolerance. Candidate genes were also found in other grasses. Nayyeripasand et al. (2021) reported *F-box* and *Na^+^/H^+^ antiporter* among their rice salt tolerance genes. Luo et al. (2019) found a double-strand break repair protein *MRE11* (GRMZM2G106056) as a salt tolerance gene in maize. Extensive lists of candidate genes have been provided by Cui et al. (2018); Liu et al. (2019); Lv et al. (2022) and Yu et al. (2018, 2023) in rice, Javid et al. (2022) in wheat, and Luo et al. (2019) in maize.

In this study, we aim to perform a genome-wide association study (GWAS) using a panel of 245 sorghum mini core accessions and 6,094,317 SNPs to identify salt tolerance genes in sorghum. We evaluated seedlings under five NaCl concentrations (0, 50, 100, 150, and 200 mmol/L), and measured shoot/root length, shoot/root fresh weight, and shoot/root dry weight. GWAS mapped 35 salt tolerance loci and we identified 39 candidate genes for salt tolerance from 29 of the 35 loci. Majority of these candidate genes (29 of the 39) have orthologs in other species that have been shown to play roles in salt tolerance in plants.

## Materials and methods

### Plant materials and salt tolerance evaluation

The mini core panel of 239 accessions (Upadhyaya et al., 2009) was used for this study. Salt (NaCl) treatments were conducted in 2019 and 2020. A total of five NaCl concentrations (0, 50, 100, 150, and 200 mmol/L) were applied in 2019, while three levels (0, 50, and 200 mmol/L) were tested in 2020 because based on 2019 results we found that 100 and 150 mmol/L didn’t provide more information. Each treatment was performed with three replicates. For each treatment, 200 plump and uniform seeds from each accession were sterilized with 0.1% mercuric chloride (HgCl_2_) solution for 15 minutes and then rinsed thoroughly three times with sterile distilled water. The sterilized seeds were placed in a germination chamber with a light intensity of 4000 lx at 30°C for 24 hours to induce germination. Germinated seeds were then sown (20 seeds per box) in germination boxes (12 cm × 12 cm) lined with two layers of moist germination paper. The germination conditions were set at 25°C with 4000 lx light for 12 hours (h) (daytime), and 20°C in darkness for 12 h (nighttime). From each replicate, ten seedlings with uniform growth were selected to measure shoot/root length (SL/RL) and shoot/root fresh weight (SFW/RFW) on the 7th day. After drying the roots and shoots at 75°C for 24 hours, shoot/root dry weight (SDW/RDW) were also measured. SL/RL were recorded to the nearest mm. SFW/RFW and SDW/RDW were measured with an analytical balance with a precision of 0.0001 g.

### Data analysis

Salt tolerance was calculated using the Seedling Tolerance Coefficient (STC) (Qiu et al., 2007; Yu et al., 2021) as follows:

STC=(value under treatment/value under control) × 100%

STC calculated for each trait was denoted with a subscript, i.e., RL_STC_ for root length STC, while root length at 50 mM or 200 mM NaCl concentrations conducted in 2020 were denoted as RL_STC_20Na_50 and RL_STC_20Na_200, respectively, and control as RL20. These totaled to 84 trait/treatment/year combinations (see **Supplementary Figure S1**; **Supplementary Table 1**). Each of the 84 traits was averaged over three replicates for GWAS.

### Association mapping

GWAS was conducted as described (Min et al., 2025). In short, GWAS for the 84 traits (**Supplementary Table S1**) was performed with 6,094,317 SNPs. We used EMMAX (Kang et al., 2010) to generate the kinship matrix (K) and STRUCTURE 2.3.4 (Pritchard et al., 2000) to calculate the Q matrix. Both were used for GWAS in an MLM model (Yu et al., 2006). Association significance threshold was based on the modified Bonferroni correction at α = 0.05, with the threshold *P* value of 8.2×10^-9^, or a −log10(*P*) value of 8.08. We also included markers with *P* value below 10^-4^ (Famoso et al., 2011; van Rooijen et al., 2015; Zhao et al., 2011) to account for association of at least three markers at a locus across more than two trait combinations to declare an association (Prinzenberg et al., 2020).

### Identification of candidate genes and haplotype analysis

The reference *Sorghum bicolor* v3.1.1 genome (McCormick et al., 2018) at Phytozome (Goodstein et al., 2012) 13 (https://phytozome-next.jgi.doe.gov/) was used to identify candidate genes. As described by Min et al. (2025), two criteria were used to find candidate genes: they either include the linked SNP or were closest to the linked SNPs. Only SNPs with <5% missing data rate were used for haplotype analysis.

### Quantitative real-time PCR analysis

qPCR was performed as previously described (Wang et al., 2025). Two salt-tolerant (IS 32787 and IS 12937) and two salt-sensitive (IS 4515 and IS 9108) mini core accessions were germinated as above. RNA was extracted from roots using the RNAprep Pure Plant Total RNA Extraction Kit (TIANGEN, Beijing, China) following the manufacturer’s instructions. ToloScript all-in-one RT EasyMix for qPCR Kit (TOLOBIO, Shanghai, China) was used to reverse transcribe mRNA into cDNA. Applied Biosystems real-time fluorescence quantitative PCR reagent (Thermo Fisher Scientific, Waltham, MA, USA) was used. The primers (**Supplementary Table S2**) were designed by QuantPrime (Arvidsson et al., 2008) with PP2A as the reference gene (Sudhakar Reddy et al., 2016). qPCR reaction was in a 20 μL volume and each sample was replicated three times. The reaction contained 5 μL cDNA, 2 μL each primer (0.1 nmol/μL), 10 μL 2 × Q3 SYBR Qpcr Master mix and 3 μL ddH_2_O. The samples were run with one cycle of 95 °C for 30 s, 40 cycles of 95°C for 10 s, 60°C for 30 s, and one cycle each of 95°C for 15 s, 60°C for 1 min, and 95°C for 15 s. Melting curves, melting temperatures and C_t_ values were analyzed with QuantStudio™ Real-Time PCR software v1.6.1, where C_t_ values were used to calculate relative expression.

## Results

### Phenotypic analysis

Salt treatments significantly reduced sorghum seedling growth. On average in both years, 50 mM NaCl treatment reduced RL by 15-16%, SL by 10-11%, and RDW by 10-13%, but SDW was only reduced by 0.5-1%, indicating that sorghum plants have basic salt tolerance; however, at 200 mM NaCl, RL was reduced by 53-54%, SL by 50-55%, RDW by 50-54%, SDW by 38-42% (**Supplementary Table S3**). Based on STC results, IS 32787 and IS 12937 were ranked as salt-tolerant and IS 4515 and IS 9108 as salt-sensitive accessions (**Supplementary Table S3**).

We calculated correlation coefficients (*r*) among the 84 trait combinations and made the following observations (**Supplementary Table S4**). 1) In both years, correlation of treatments with control decreased as the salt levels increased for RDW19, RFW19, RL19, SDW19, SFW19, SL19, RFW20, RL20, SFW20, and SL20, with *r* ranging from 0.48-0.91. 2) There was also significant positive correlation between RL19 and RFW19 (*r* = 0.43-0.82), RDW19 and SDW19 (*r* = 0.42-0.67), and SL19 and SFW19 (*r* = 0.35-0.88) within each treatment. 3) Phenotypic values among the treatment within the same trait were also significantly correlated for RDW19, RL19, SFW19, SL19, RFW20, RL20, SFW20, and SL20. 4) RDW and SDW were highly correlated (*r* = 0.42-0.67) within treatments, indicating some degree of growth coordination between shoots and roots. 5) Both RL_STC_ and SL_STC_ were highly and positively correlated (*r* = 0.63-0.83). These suggest that growth under control condition was highly predictable of growth under salt stress treatment, but this predictability diminished slightly with increasing salt stress.

### GWAS mapping

We mapped 35 salt tolerance related loci, each represented by multiple SNP markers; 18 of the 35 were STC traits. Among the 35 loci, all except one (10-4) were detected in at least one other experiment (control or treatment), indicating the mapped loci were stable across experiments in the same year. The locus with strongest [highest -log(*P*) value] association was 8–1 with -log(*P*) value ranging from 35.8323 to 48.6724. Loci 1-3, 1-4, and 7–2 were each detected across six treatment/trait combinations while 3-4, 3-5, 4-6, 6-1, 7-1, 9–2 were each detected across five treatment/trait combinations; the rest were detected between 2–4 treatment/trait combinations. Among the 35 loci, 14 were pleiotropic for more than one trait while the other 21 were for single growth traits, six for RDW, five for SDW, four for SFW and three each for SL and RL. Two RFW loci (1–3 and 10-4) were pleiotropic with other traits. One pleiotropic locus (4-6) was mapped to the control (RDW19) and all four salt treatments (RDW19Na_50, RDW19Na_100, RDW19Na_150, RDW19Na_200) (**Supplementary Table S8**).

### Candidate gene identification

In 29 of the 35 loci, we identified 39 candidate genes for salt tolerance (**Supplementary Table S8**). Majority of these genes (29 of the 39) have orthologs in other species that have been shown to play roles in salt tolerance (see Discussion). Three of these genes, in Loci 2-7, 6–2 and 7-1, were sorghum-specific genes with unknown functions. There were three F-box containing proteins in Loci 2-1, 3–4 and 4-3, respectively (**Supplementary Table S8**). We found 21 of the 39 genes contained linked SNPs in either its proximal promoter, coding or 3’-UTR. Among eight cases where SNP(s) was located in exon(s), in two cases it caused synonymous mutation but non-synonymous in the other six cases. In the remaining 13 cases, these SNPs were either located in introns (Loci 1-4, two genes in 3-2, 4-6, 7-2, 9–3 and 10-1), 3’-UTRs [Loci 2-6 (two genes), 10–2 and 10-4] or promoters (Loci 4–1 and 4-4) (**Table 1**).

**Table 1:** Location of linked SNPs in genic regions in sorghum.

Locus	Gene ID	SNP annotation
1-4	Sobic.001G431400	71037460/71039135 in introns 2, 5
	Sobic.001G431500	71047857/71048187 in exon but both synonymous
2-5	Sobic.002G393100	74592425 in exon 6 is non-synonymous; all others in the second intron
2-6	Sobic.002G421300	all in 3’-UTR
	Sobic.002G421350	all in introns or 3’-UTR
3-1	Sobic.003G019900	1750689/1750699 in exon 2 (see text)
3-2	Sobic.003G021500	1838250 in intron 1
	Sobic.003G021600	1846262 and 1855323 both in introns
	Sobic.003G021700	1860237 in exon 2 is nonsynonymous
4-1	Sobic.004G003900	526 bp upstream from coding region
4-2	Sobic.004G008300	733965 in exon 2 is synonymous
4-4	Sobic.004G101900	407 bp upstream from coding region
4-6	Sobic.004G264000	60885024 in intron 1
6-1	Sobic.006G064700	42467059 in exon 3 is non-synonymous; all others in introns or 3’-UTR
7-2	Sobic.007G059400	all in introns
9-2	Sobic.009G015166	1349130 UAG is a stop codon; 1349692 is synonymous; all others are in introns
	Sobic.009G015200	1384724 in exon 7; 1386013 in exon 6; both non-synonymous
9-3	Sobic.009G211200	all in intron 8
10-1	Sobic.010G025850	all in intron 7
10-2	Sobic.010G162500	all in 3’-UTR
10-4	Sobic.010G234400	57710773 in 3’-UTR

### Gene expression and qPCR

Candidate gene expression based on data available from GeneAtlas v2 FPKM (McCormick et al., 2018) is provided in **Supplementary Table S5**. Nine of the 39 candidate genes from eight loci showed root-preferential expression: *Sobic.003G021600* (3-2; *ATP-dependent RNA helicase DDX35*), *Sobic.004G008300* (4-2; *fatty acid hydroxylase*), *Sobic.004G264000* (4-6; *receptor protein kinase*), *Sobic.007G050700* (7-1; *unknown protein*), *Sobic.007G059400* (7-2; *anthocyanin 5-aromatic acyltransferase*), *Sobic.009G015166* (*DUF247*) and *Sobic.009G015200* (*unknown protein*) (9-2), *Sobic.009G256800* (9-4; *unknown protein*), and *Sobic.010G234400* (10-4; *O-methyltransferase ZRP4*).

**Figure 1:**
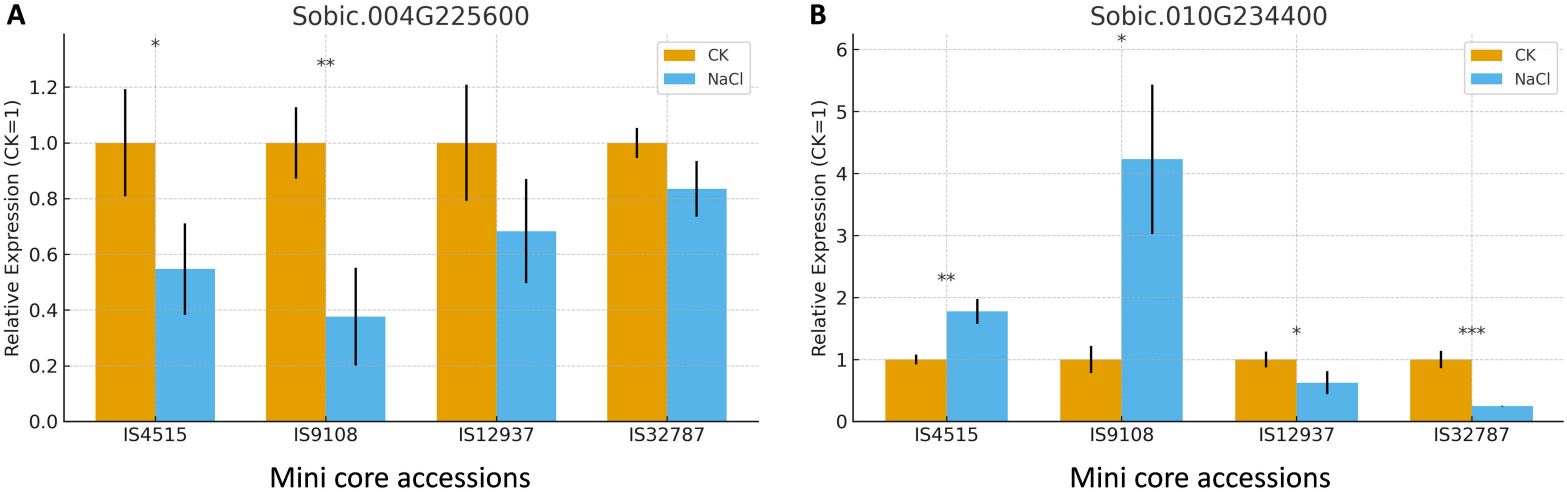
qPCR of *Sobic.004G225600***(A)** and *Sobic.010G234400***(B)** from roots of two salt-tolerant (IS 12937, IS 32787) and two salt-sensitive (IS 4515, IS 9108) mini core accessions. CK: control. NaCl: 200 mM NaCl treatment. *, **, *** indicate significant differences with control at P < 0.05, 0.01 and 0.001, respectively.

We selected eight candidate genes for qPCR analysis: *Sobic.001G038601* (*unknown*), *Sobic.003G207600* (*F-box domain*), *Sobic.004G225600* (*DEAD-box ATP-dependent RNA helicase 47A*), *Sobic.004G264000* (*receptor protein kinase*), *Sobic.004G264100* (*Na^+^/H^+^ antiporter*), *Sobic.007G059400* (*anthocyanin 5-aromatic acyltransferase*), *Sobic.010G025850* (*Myeloid leukemia factor 1-interacting protein*), and *Sobic.010G234400* (*O-methyltransferase ZRP4*). Two of these genes, *Sobic.003G207600* and *Sobic.004G264100*, failed. For *Sobic.004G225600*, salt treatment down-regulated its expression in both the tolerant and sensitive accessions upon salt treatment; the only difference was that its down-regulation was significant in sensitive accessions (**Figure 1A**). On the other hand, *Sobic.010G234400*’s expression was significantly down-regulated in salt-tolerant accessions but up-regulated in salt-sensitive accessions (**Figure 1B**). The expression of the other four genes was less consistent (**Supplementary Figure S2**).

### Haplotype analysis

**Figure 2:**
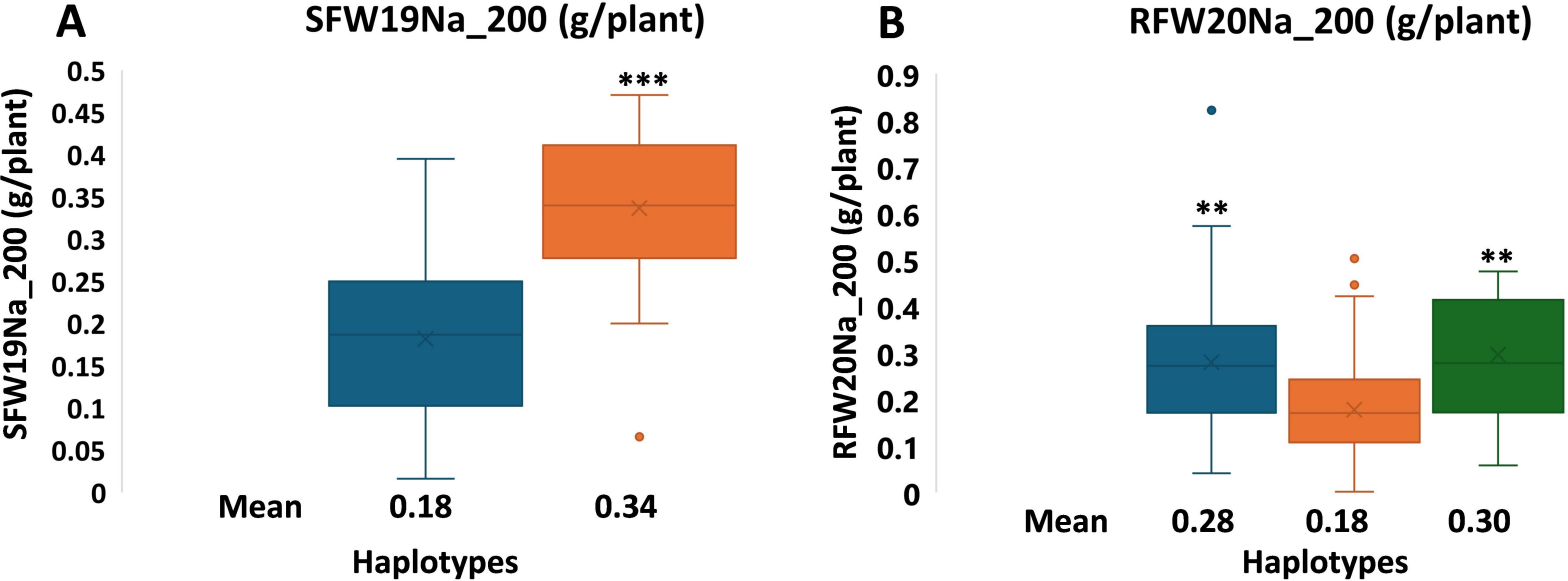
Haplotypic effects of Loci 4–5 and 10–4 on SFW19Na_200 **(A)** and RFW20Na_200 **(B)**, respectively. In both **(A, B)**, from left to right are Haplotypes I to III and *P* values were based on comparison to the lowest haplotypes. The × inside each box in the boxplot represents the mean and the horizontal line represents the median value of each data group. ** and *** indicates difference from the lowest was at *P* < 0.01 and 0.001, respectively, using *t*-test.

We performed haplotype analysis with SNPs from Locus 4–5 for SDW19Na_200 and 10–4 for RFW20Na_200. The four SNPs from Locus 4-5, 57576627, 57576638, 57576654 and 57576672, were all located between *Sobic.004G225600* and *Sobic.004G225700* (*DNA REPAIR PROTEIN RAD7*). They fell into two haplotypes (**Table 2**), including 100 Haplotype Is, and 22 IIs. Their SFW (SFW19Na_200) averaged 0.1817 and 0.3366 g/plant, respectively and Haplotype II with 0.3366 average SFW were significantly higher than that of Haplotype I’s average of 0.1817 (*P* < 1.36 × 10^-7^) (**Figure 2A**) and all top seven SFW accessions were Haplotype II (**Supplementary Table S6**). Similarly, among the three SNPs from Locus 10-4, 57710585, 57710592 and 57710613 were all in the 3’-UTR of Sobic.010G234300 (unknown protein) which overlaps with the 3’-UTR of *Sobic.010G234400* (*O-methyltransferase ZRP4*). The three also fell into three haplotypes (**Table 3**). In this locus, Haplotypes I, II and III had 45, 181, and 7 accessions and their RFW (RFW20Na_200) averaged 0.28, 0.18 and 0.30, respectively. Again, Haplotypes I and III accessions had significantly higher RFW than those of II (**Figure 2B**) and all top four RFW accessions were of Haplotype I (**Supplementary Table S7**). This information may be useful in marker-assisted selection in sorghum breeding.

**Table 2:** Two haplotypes of SNPs from Locus 4-5 (see **Figure 2A**).

SNP Haplotype	57576627	57576638	57576654	57576672
Haplotype I	A	A	C	C
Haplotype II	M	R	Y	Y

**Table 3:** Three haplotypes of SNPs from Locus 10-4 (see **Figure 2B**).

SNP Haplotype	57710585	57710592	57710613
Haplotype I	A	G	G
Haplotype II	G	T	T
Haplotype III	R	K	K

## Discussion

In this study, we mapped seedling salt tolerance of the sorghum mini core collection to 35 loci. Interestingly, the most tightly associated locus 8–1 is located in a non-coding region (no protein-coding genes are annotated within 80 kb regions on each side of the locus). Causal variants identified by GWAS in non-coding regions are not uncommon, especially in humans (Alsheikh et al., 2022). These non-coding regions may impact the phenotype by altering enhancers, transcription factor binding sites, chromatin state (Alsheikh et al., 2022; Schipper and Posthuma, 2022) or microRNA expression (Božić et al., 2024). Future projects will investigate these possibilities. But for the rest of the loci, 29 of the 39 candidate genes have been found to have orthologs in other species. In the following sections, we outline their roles in plant salt tolerance.

Response of gene network to salt in sorghum has been elegantly presented by Amombo et al. (2022) and many candidate genes reported in this study may play a role in sorghum salt tolerance in this network. Salt immediately causes three problems: osmotic stress, cytotoxic ROS accumulation and Na^+^/Cl^-^ toxicity (Amombo et al., 2022; van Zelm et al., 2020). But before all these are started, plants need to sense the salt presence. This seems to be carried out by receptor-like kinases (RLKs) (van Zelm et al., 2020). For example, RLK can sense the salt-induced changes in the cell wall (Feng et al., 2018). We found an *RLK* gene (*Sobic.004G264000*) from Locus 4–6 that may play this role in sorghum. Plants confront ROS by activating antioxidant genes to scavenge excess ROS (Amombo et al., 2022) and if the ROS is not maintained at normal level, it may cause double-stranded DNA break (Zvanarou et al., 2020). *DNA REPAIR PROTEIN RAD7* from Locus 4–5 may be an enzyme for such DNA repair. *Myeloid leukemia factor 1-interacting protein* (*MLF*) in Locus 10–1 may be related to ROS as stressors induce both ROS production and *MLF* expression (Wu et al., 2023). Fatty acid hydroxylases such as the one found in Locus 4–2 enhance salt tolerance by modulating fatty acid metabolic pathways and improving cell membrane stability and antioxidant capacity (Gu et al., 2025). Flavonoids including anthocyanins are powerful antioxidants to scavenge ROS (Huchzermeyer et al., 2022) and have been demonstrated to play a major role in sorghum salt tolerance (Ren et al., 2022). This is why anthocyanin synthesis related genes such as *anthocyanin 5-aromatic acyltransferase* (Murayama et al., 2021) from Locus 7–2 and *HOMEOBOX-LEUCINE ZIPPER PROTEIN ANTHOCYANINLESS 2* (Kubo et al., 1999) from Locus 3–3 can be induced by salt treatment (Wang et al., 2023). Related to ROS, *Sobic.010G162500* (*CO dehydrogenase flavoprotein-like, FAD-binding*) in Locus 10–2 is ortholog to *AT4G20860* (*BBE22*) involved in H_2_O_2_ generation (Santamaría et al., 2018). *O-methyltransferase* (Locus 10-4) (Hafeez et al., 2021) and *flavonol synthase* from Locus 3–2 are involved in flavonoid synthesis and overexpressing flavonol synthases in plants increases tolerance to saline-alkali stress by enhancing flavonol accumulation, antioxidant capacity and osmotic balance (Zhang et al., 2023) and increases K^+^/Na^+^ ratio which in turn leads to salt tolerance (Wang et al., 2021). Therefore, the flavonoid pathway may also be relevant in maintaining K^+^/Na^+^ homeostasis to minimize Na^+^/Cl^-^ toxicity (Amombo et al., 2022; Ren et al., 2022). Similarly, overexpressing sorghum *Na^+^/H^+^ antiporter* (one found in Locus 4-6) also promoted antioxidative enzyme activities, lower Na^+^ and higher Ca^2+^ levels in roots which led to Na^+^ exclusion and increased salt tolerance (Kumari et al., 2017). Also playing a role in maintaining K^+^/Na^+^ homeostasis are response regulators such as the one found in Locus 6-1, *Sobic.006G064700* (*RESPONSE REGULATORY DOMAIN-CONTAINING PROTEIN*), which is orthologous to Arabidopsis AT2G25180 (*ARR12*). ARR12 regulates Na^+^ accumulation in the shoots by controlling the expression of *AtHKT1;1* (HKT: high-affinity K^+^ transporter) in the roots (Mason et al., 2005) and HKT is also central to alleviating Na^+^/Cl^-^ toxicity (Amombo et al., 2022). Na^+^/Ca^2+^ exchanger NCL such as the one in Locus 3–2 transport Ca^2+^ to the cytosol and sequester cytosolic Na^+^ into the vacuole; consequently, Arabidopsis *atncl* mutant is sensitive to salt stress (Ishu et al., 2025; Wang et al., 2012). Also affecting the K^+^/Na^+^ homeostasis is nitric-oxide (NO) synthase (ortholog found in Locus 4-1) as its mutation produces greater Na^+^/K^+^ ratio in shoots due to enhanced Na^+^ and reduced K^+^ accumulation when exposed to salt (Zhao et al., 2007). Brassinosteroid (BR) biosynthesis genes can be induced by salt and BR-mediated S-nitrosoglutathione reductase negatively regulates NO to reduce ROS and induce genes related to Na^+^ and K^+^ transport, leading to the decrease of Na^+^/K^+^ ratio in the roots (Zeng et al., 2024). *Sobic.002G393100* (*estrogen 17-oxidoreductase*) in Locus 2–6 is orthologous to *AT5G50700* (*AtHSD1*; HSD: 11-β-hydroxysteroid dehydrogenase) and plants overexpressing *AtHSD1* constitutively expressed BR response genes (Li et al., 2007), linking this gene to salt tolerance through BR. Citrate synthase (Liang et al., 2021) (ortholog in Locus 4-4) and small subunit ribosomal protein S3Ae (Liang et al., 2015) (ortholog in Locus 1-4) provide salt tolerance through tolerance to osmotic stress.

Other candidate genes provide salt tolerance through other/unknown pathways not included by Amombo et al. (2022). α/β-Hydrolases (ortholog in Locus 1-4) include many members and some have been shown to enhance salt tolerance (Liu et al., 2014). Another large gene family is F-box proteins (orthologs found in Loci 2-1, 3-4, and 4-3) and again some members are induced by salt (Jia et al., 2017) or confer salt tolerance (Xu et al., 2014). *RICIN B-LIKE LECTIN R40G3* in Locus 2–7 is orthologous to *LOC_Os07g48500* (*Osr40g3*) which improves salt tolerance (Roy et al., 2025). GDSL lipase/esterase (ortholog in Locus 3-1) in Arabidopsis, *AtLTL1*, increases salt tolerance when overexpressed (Naranjo et al., 2006). ATP-dependent RNA helicases (found in Loci 3–2 and 4-5) can be salt-induced (Nakamura et al., 2004) or increase salt tolerance when overexpressed (Nguyen et al., 2018). *Heading date 5* in 7–2 is orthologous to AT4G14540/NF-YB3 and overexpression of *PwNF-YB3* in Arabidopsis showed a significant tolerance to salinity, drought and osmotic stress (Zhang et al., 2015). *tRNA(His) guanylyltransferase* in Locus 9–3 is orthologous to rice *LOC_Os05g45890*/*AET1* whose mutation causes sensitivity to both salt and drought (Chen et al., 2019).

In summary, we conducted a GWAS using a panel of 245 sorghum mini core accessions and 6,094,317 SNPs. We evaluated seedlings under five NaCl concentrations (0, 50, 100, 150, and 200 mmol/L) in 2019 and three (0, 50, and 200 mmol/L) in 2020 for shoot/root length, shoot/root fresh weight, and shoot/root dry weight, resulting in 84 trait/treatment/year combinations. GWAS of these 84 combinations mapped 35 loci, 39 candidate genes were identified from 29 of the 35 loci and 29 of the 39 have orthologs in other species that have been shown to play roles in salt tolerance in plants. These candidate genes include those potentially involved in K^+^/Na^+^ homeostasis such as O-methyltransferase (Locus 10-4), flavonol synthase (Locus 3-2), Na^+^/H^+^ antiporter (Locus 4-6), response regulators (Locus 6-1, Na^+^/Ca^2+^ exchanger NCL (Locus 3-2), nitric-oxide synthase (Locus 4-1) and estrogen 17-oxidoreductase/11-β-hydroxysteroid dehydrogenase (Locus 2-6). Other genes are potentially related to stress signaling and growth regulation were also identified in genomic regions in or adjacent to the location of associated markers. These findings provide genes for functional studies and markers for molecular breeding.

## Data Availability

The original contributions presented in the study are included in the article/**Supplementary Material**. Further inquiries can be directed to the corresponding author.

## Supplementary material

Supplementary Figure 1 Genmone-wide association analysis of 84 Traits for Salt Stress Manhattan plots.

Supplementary Figure 2 qPCR of four selected candidate genes *Sobic.001G038601***(A)**, *Sobic.004G264000***(B)**, *Sobic.007G059400***(C)** and *Sobic.010G025850***(D)** from roots *in two* salt-tolerant (IS12937, IS32787) and two salt-sensitive (IS4515, IS9108) accessions. CK: control. NaCl: 200 mM NaCl treatment. *, **, indicate significant differences with control at P < 0.05 and 0.01, respectively.
