# Supplementary figures and images for: Genome-wide association study of salt tolerance in sorghum during germination
Source: Front Plant Sci. 2025 Dec 10;16:1682270. doi: 10.3389/fpls.2025.1682270 (PMC12728437; doi:10.3389/fpls.2025.1682270)

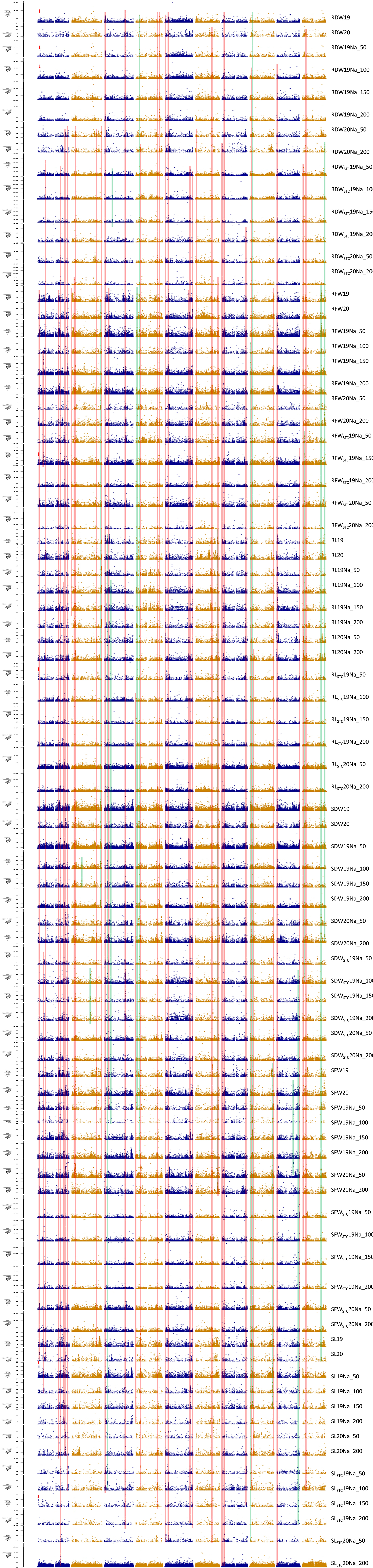

Supplement: Supplementary Figure 1 — Genmone-wide association analysis of 84 Traits for Salt Stress Manhattan plots. [file Image1.jpeg]

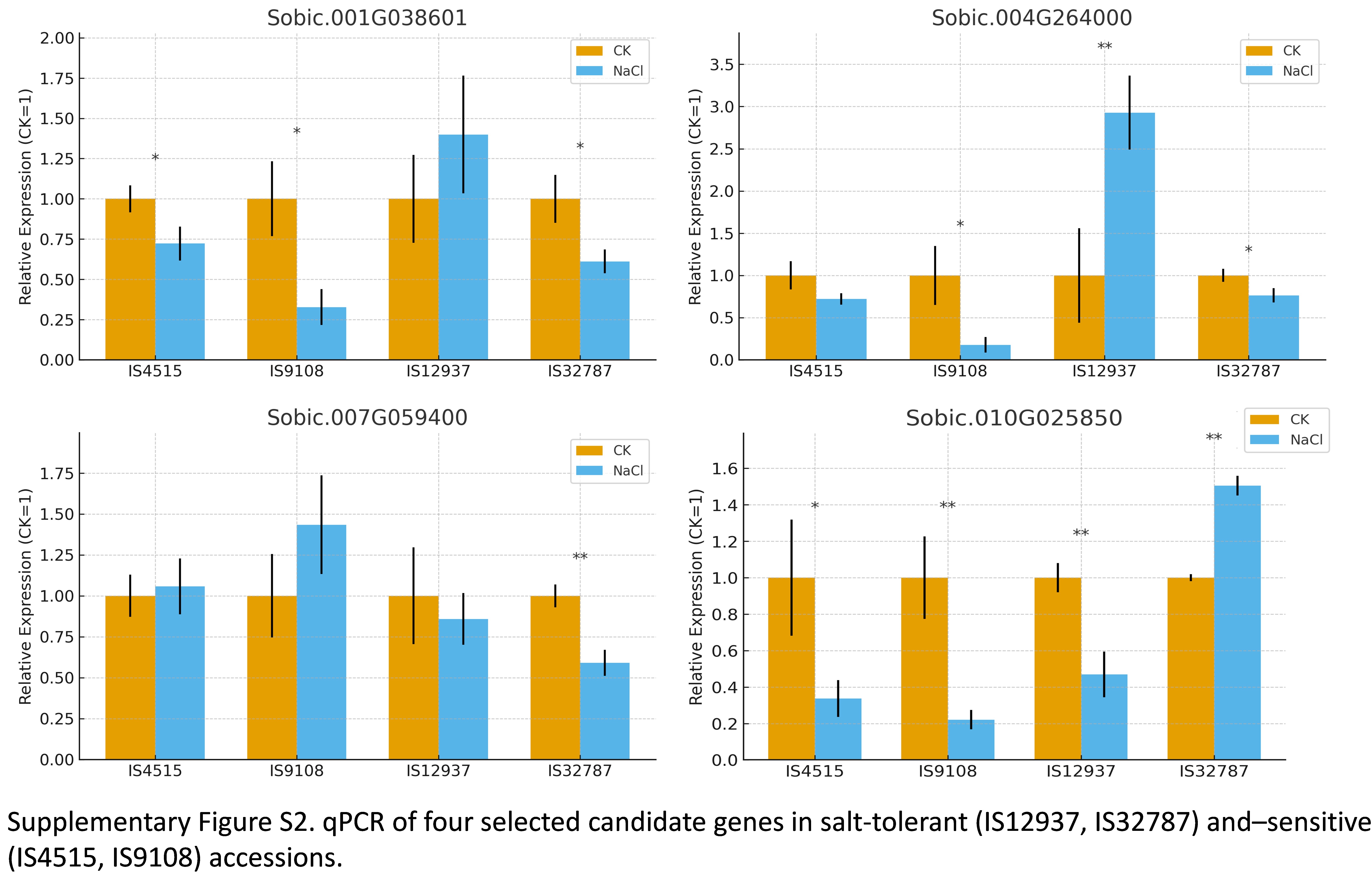

Supplement: Supplementary Figure 2 — qPCR of four selected candidate genes Sobic.001G038601(A), Sobic.004G264000(B), Sobic.007G059400(C) and Sobic.010G025850(D) from roots in two salt-tolerant (IS12937, IS32787) and two salt-sensitive (IS4515, IS9108) accessions. CK: control. NaCl: 200 mM NaCl treatment. *, **, indicate significant differences with control at P < 0.05 and 0.01, respectively. [file Image2.jpeg]
