# Supplementary material for: Genome-wide association study of salt tolerance in sorghum during germination
Source: Front Plant Sci. 2025 Dec 10;16:1682270. doi: 10.3389/fpls.2025.1682270 (PMC12728437; doi:10.3389/fpls.2025.1682270)
Supplement: Supplementary file 4 [file Supplementaryfile2.docx]

**Supplementary Tables S8**. Salt tolerance related loci and their associated candidate genes in sorghum*.

| Locus | Top SNPs | Trait/-log(P) value | | | | | | Candidate Gene |
| --- | --- | --- | --- | --- | --- | --- | --- | --- |
| 1-1 |  | SFW_STC_19Na_50 | SFW_STC_19Na_150 | SFW_STC_19Na_200 |  |  |  | Sobic.001G038601  Chr01:2891570..2892074 forward  unknown  not sorghum-specific |
|  | 2894425 | 7.6148 | 6.6664 | **8.4481** |  |  |  |  |
|  | 2894439 | 7.4922 | 6.6041 | **8.3808** |  |  |  |  |
|  | 2894444 | **10.4949** | **9.9551** | **12.0991** |  |  |  |  |
|  | 2894445 | **10.5245** | **10.0067** | **12.1348** |  |  |  |  |
|  |  |  |  |  |  |  |  |  |
| 1-2 |  | SDW_STC_19Na_150 | SDW_STC_19Na_200 |  |  |  |  |  |
|  | 39686977 | **10.1757** | **9.5466** |  |  |  |  |  |
|  | 39686983 | 5.3039 | 5.3341 |  |  |  |  |  |
|  | 39686998 | 5.3167 | 5.3494 |  |  |  |  |  |
|  |  |  |  |  |  |  |  |  |
| 1-3 |  | RDW_STC_20Na_50 | RDW_STC_20Na_200 | RFW_STC_20Na_200 | RL_STC_20Na_200 | SDW_STC_20Na_200 | SL_STC_20Na_200 |  |
|  | 59255607 | 7.5433 | **15.9932** | **16.1597** | **18.3433** | 7.0703 | **13.2051** |  |
|  | 59255631 | 5.3994 | **12.2299** | **12.8464** | **11.9832** | 6.8109 | **10.6147** |  |
|  | 59255648 | 7.4336 | **16.2010** | **16.2138** | **18.4632** | 7.1573 | **13.2846** |  |
|  |  |  |  |  |  |  |  |  |
| 1-4 |  | SDW20 | SDW20Na_50 | SFW20 | SFW20Na_50 | SL20Na_50 | SL20Na_200 | Sobic.001G431400  Chr01:71037018..71039276 forward  small subunit ribosomal protein S3Ae  Sobic.001G431500  Chr01:71047781..71048900 reverse  alpha/beta-Hydrolases superfamily |
|  | 71034060 | 6.1928 | 7.5154 | 5.8785 | 7.1840 | 5.4345 | 4.3654 |  |
|  | 71034875 | 6.7294 | 7.7406 | 6.1523 | 6.8848 | 4.8357 | 4.0647 |  |
|  | 71035060 | 4.5295 | 5.3359 | 5.4881 | 8.0531 | 5.8113 | 6.2481 |  |
|  |  |  |  |  |  |  |  |  |
|  | 71040898 | 5.5084 | 6.3998 | 5.9617 | 6.8650 | 5.7193 | 4.6002 |  |
|  | 71041801 | 5.4246 | 7.0955 | 5.3033 | 7.0419 | 5.7665 | 4.5814 |  |
|  | 71044200 | 3.9638 | 5.1169 | 5.5229 | 7.0864 | 6.7507 | 6.7228 |  |
|  |  |  |  |  |  |  |  |  |
| 2-1 |  | SFW20 | SFW20Na_50 | SDW20 |  |  |  | Sobic.002G089900  Chr02:9404292..9416619 forward  F-BOX DOMAIN-CONTAINING PROTEIN |
|  | 9397093 | 7.8863 | 7.6809 | **8.4206** |  |  |  |  |
|  | 9399803 | 7.3082 | 7.3663 | 7.6386 |  |  |  |  |
|  | 9400839 | 7.2913 | 7.4611 | 7.4923 |  |  |  |  |
|  | 9401517 | 6.2586 | 7.7060 | 6.3321 |  |  |  |  |
|  | 9403420 | 7.4296 | 7.4524 | 7.6322 |  |  |  |  |
|  | 9404018 | 6.4416 | 6.6228 | 6.1553 |  |  |  |  |
|  |  |  |  |  |  |  |  |  |
| 2-2 |  | SFW_STC_19Na_150 | SFW_STC_19Na_200 |  |  |  |  |  |
|  | 10416294 | **11.5527** | **14.0996** |  |  |  |  |  |
|  | 10416303 | **11.4080** | **13.9458** |  |  |  |  |  |
|  | 10416367 | 7.2502 | **8.6468** |  |  |  |  |  |
|  |  |  |  |  |  |  |  |  |
| 2-3 |  | SDW19 | SDW19Na_50 |  |  |  |  |  |
|  | 10825030 | 5.3586 | 6.0967 |  |  |  |  |  |
|  | 10825041 | 5.6301 | 5.7289 |  |  |  |  |  |
|  | 10825043 | 5.6301 | 5.7289 |  |  |  |  |  |
|  |  |  |  |  |  |  |  |  |
| 2-4 |  | SDW_STC_19Na_100 | SDW_STC_19Na_200 |  |  |  |  |  |
|  | 47909235 | 7.0760 | 6.5557 |  |  |  |  |  |
|  | 47909412 | 7.0796 | 6.6661 |  |  |  |  |  |
|  | 47909450 | 7.1056 | 6.6465 |  |  |  |  |  |
|  |  |  |  |  |  |  |  |  |
| 2-5 |  | SDW19 | SDW19Na_50 | SDW19Na_100 | SDW19Na_200 |  |  | Sobic.002G393100  Chr02:74592119..74594652 reverse  estrogen 17-oxidoreductase |
|  | 74593910 | 5.5902 | 5.2156 | 5.5044 | 5.7135 |  |  |  |
|  | 74593973 | 5.6424 | 5.2393 | 5.5399 | 5.5820 |  |  |  |
|  | 74594001 | 5.5902 | 5.2156 | 5.5044 | 5.7135 |  |  |  |
|  | 74594023 | 5.7961 | 5.3107 | 5.6219 | 5.8509 |  |  |  |
|  | 74594030 | 5.7961 | 5.3107 | 5.6219 | 5.8509 |  |  |  |
|  |  |  |  |  |  |  |  |  |
| 2-6 |  | RL19Na_50 | RL19Na_200 |  |  |  |  | Sobic.002G421300  Chr02:76860175..76861505 forward  RICIN B-LIKE LECTIN R40G3  Sobic.002G421350  Chr02:76875264..76876357 forward  unknown---sorghum-specific |
|  | 76861464 | 5.5619 | 4.5541 |  |  |  |  |  |
|  | 76866527 | 5.9541 | 5.0054 |  |  |  |  |  |
|  | 76873040 | 5.1183 | 5.1702 |  |  |  |  |  |
|  | 76875475 | 5.8751 | 5.4779 |  |  |  |  |  |
|  | 76875599 | 5.8530 | 5.5243 |  |  |  |  |  |
|  | 76876018 | 5.6328 | 5.3202 |  |  |  |  |  |
|  |  |  |  |  |  |  |  |  |
| 3-1 |  | SL19Na_50 | SL19Na_100 |  |  |  |  | Sobic.003G019900  Chr03:1749599..1753721 forward  GDSL lipase/esterase |
|  | 1750689 | 3.9421 | 6.0706 |  |  |  |  |  |
|  | 1750699 | 5.0368 | 6.4355 |  |  |  |  |  |
|  | 1750781 | 5.0811 | 6.0573 |  |  |  |  |  |
|  |  |  |  |  |  |  |  |  |
| 3-2 |  | RDW19 | RDW19Na_50 | RDW19Na_200 |  |  |  | Sobic.003G021500  Chr03:1835933..1839841 reverse  SODIUM_CALCIUM EXCHANGER NCL1  Sobic.003G021600  Chr03:1845893..1858015 reverse  ATP-dependent RNA helicase DDX35  Sobic.003G021700  Chr03:1859528..1860643 forward  flavonol synthase |
|  | 1834466 | 5.1526 | 5.2850 | 5.5674 |  |  |  |  |
|  | 1838250 | 5.1380 | 5.3828 | 5.7379 |  |  |  |  |
|  | 1840336 | 5.3953 | 5.6879 | 5.8962 |  |  |  |  |
|  | 1844505 | 5.1526 | 5.2850 | 5.5674 |  |  |  |  |
|  | 1846262 | 5.4118 | 5.6930 | 5.8882 |  |  |  |  |
|  | 1855323 | 5.4118 | 5.6930 | 5.8882 |  |  |  |  |
|  | 1860237 | 5.1526 | 5.2850 | 5.5674 |  |  |  |  |
|  | 1861374 | 5.4118 | 5.6930 | 5.8882 |  |  |  |  |
|  | 1863181 | 6.0863 | 6.1312 | 5.8702 |  |  |  |  |
|  |  |  |  |  |  |  |  |  |
| 3-3 |  | SL_STC_19Na_50 | SL_STC_19Na_100 |  |  |  |  | Sobic.003G096100  Chr03:8499491..8501691 reverse  EamA-like transporter  Sobic.003G096300  Chr03:8516800..8523922 reverse  HOMEOBOX-LEUCINE ZIPPER PROTEIN ANTHOCYANINLESS 2 |
|  | 8505195 | 5.1129 | 5.3808 |  |  |  |  |  |
|  | 8505602 | 5.3580 | 5.6591 |  |  |  |  |  |
|  | 8506794 | 5.0803 | 5.4951 |  |  |  |  |  |
|  | 8508616 | 5.0803 | 5.4951 |  |  |  |  |  |
|  | 8508721 | 5.2817 | 5.5643 |  |  |  |  |  |
|  | 8508782 | 5.0910 | 5.6262 |  |  |  |  |  |
|  | 8509330 | 7.1088 | 5.7640 |  |  |  |  |  |
|  | 8509794 | 6.5554 | 6.4982 |  |  |  |  |  |
|  | 8509797 | 5.1492 | 6.2903 |  |  |  |  |  |
|  |  |  |  |  |  |  |  |  |
| 3-4 |  | RDW19 | RDW19Na_50 | SDW19 | SDW19Na_100 | SDW19Na_150 |  | Sobic.003G207600  Chr03:53877624..53879961 forward  F-box domain |
|  | 53869864 | **8.2985** | **10.5772** | 6.5273 | 6.8342 | 6.9700 |  |  |
|  | 53869870 | **8.3576** | **10.6558** | 6.6195 | 6.8378 | 7.0124 |  |  |
|  | 53869871 | **8.3111** | **10.6216** | 6.5565 | 6.7874 | 6.9808 |  |  |
|  | 53869879 | **8.2593** | **10.5266** | 6.6375 | 6.7831 | 6.9887 |  |  |
|  |  |  |  |  |  |  |  |  |
| 3-5 |  | SL_STC_19Na_100 | SDW_STC_19Na_100 | SDW_STC_19Na_150 | SL_STC_19Na_150 | SL_STC_19Na_200 |  | Sobic.003G209900  Chr03:54219403..54220470 forward  Chlorophyll a-b binding protein 1 |
|  | 54216235 | 5.8264 | **8.0973** | 7.8334 | 6.6123 | 6.0348 |  |  |
|  | 54216253 | 6.2324 | **8.4194** | **9.0798** | 7.9175 | 7.2561 |  |  |
|  | 54216368 | 5.0215 | 6.4390 | 8.0273 | 6.6147 | 5.9772 |  |  |
|  |  |  |  |  |  |  |  |  |
|  |  | SL19Na_150 | SL19Na_200 |  |  |  |  | Sobic.004G003900  Chr04:346783..352642 forward  nitric-oxide synthase, plant (NOA1) |
| 4-1 | 346203 | 5.9794 | 7.2656 |  |  |  |  |  |
|  | 346219 | 4.5774 | 5.4446 |  |  |  |  |  |
|  | 346257 | 5.2698 | 5.5373 |  |  |  |  |  |
|  |  |  |  |  |  |  |  |  |
| 4-2 |  | RL_STC_19Na_100 | RL_STC_19Na_150 | RL_STC_19Na_200 |  |  |  | Sobic.004G008300  Chr04:733524..734761 reverse  Fatty acid hydroxylase |
|  | 729832 | 5.5179 | **9.6940** | 5.4478 |  |  |  |  |
|  | 729843 | 5.5893 | **9.7396** | 5.5441 |  |  |  |  |
|  | 729857 | 5.3554 | **9.2180** | 5.2402 |  |  |  |  |
|  | 733965 | 5.3220 | 5.2787 | 2.7201 |  |  |  |  |
|  |  |  |  |  |  |  |  |  |
| 4-3 |  | SDW_STC_19Na_50 | SL_STC_19Na_50 | SL_STC_19Na_100 |  |  |  | Sobic.004G118300  Chr04:12476897..12479996 reverse  F-BOX DOMAIN CONTAINING PROTEIN |
|  | 12469437 | 6.2934 | 6.6221 | 5.6037 |  |  |  |  |
|  | 12469637 | 6.3360 | 6.4571 | 5.5203 |  |  |  |  |
|  | 12469642 | 6.2662 | 6.1242 | 5.2740 |  |  |  |  |
|  | 12469648 | 6.1857 | 6.0439 | 5.3176 |  |  |  |  |
|  | 12469716 | 5.8177 | 6.1095 | 5.0485 |  |  |  |  |
|  | 12469737 | 6.1653 | 6.3013 | 5.5175 |  |  |  |  |
|  |  |  |  |  |  |  |  |  |
| 4-4 |  | SDW_STC_19Na_50 | SDW_STC_19Na_100 | SDW_STC_19Na_200 |  |  |  | Sobic.004G101900  Chr04:9417186..9422351 forward  Citrate synthase |
|  | 9416752 | **10.2137** | 6.1546 | 5.4997 |  |  |  |  |
|  | 9416758 | **10.3823** | 6.1021 | 5.5702 |  |  |  |  |
|  | 9416779 | **10.3067** | 5.9324 | 5.3621 |  |  |  |  |
|  |  |  |  |  |  |  |  |  |
| 4-5 |  | SL19Na_200 | SFW19Na_150 | SFW19Na_200 | SDW19Na_200 |  |  | Sobic.004G225600  Chr04:57570425..57573539 reverse  DEAD-box ATP-dependent RNA helicase 47A  Sobic.004G225700  Chr04:57577912..57580393 reverse  DNA REPAIR PROTEIN RAD7 |
|  | 57576627 | 5.5754 | 6.1352 | **8.6821** | 5.4736 |  |  |  |
|  | 57576638 | 5.5902 | 5.1765 | **8.2023** | 5.5546 |  |  |  |
|  | 57576654 | 5.7819 | 5.2433 | **8.1876** | 6.0508 |  |  |  |
|  | 57576672 | 5.6107 | 5.5778 | **8.6207** | 6.1209 |  |  |  |
|  |  |  |  |  |  |  |  |  |
| 4-6 |  | RDW19 | RDW19Na_50 | RDW19Na_100 | RDW19Na_150 | RDW19Na_200 |  | Sobic.004G264000  Chr04:60882520..60885416 reverse  Receptor protein kinase  Sobic.004G264100  Chr04:60889687..60892610 reverse  Na^+^/H^+^ antiporter |
|  | 60885024 | 3.7500 | 5.3376 | 4.2110 | 4.6412 | 7.6636 |  |  |
|  | 60888623 | 5.0032 | 6.6779 | 5.5770 | 5.8829 | **9.5385** |  |  |
|  | 60889135 | 6.0659 | 7.9024 | 6.7797 | 7.3702 | **12.1359** |  |  |
|  |  |  |  |  |  |  |  |  |
| 5-1 |  | RDW_STC_19Na_50 | RDW_STC_19Na_100 | RDW_STC_19Na_150 |  |  |  |  |
|  | 57913503 | 6.3105 | 5.5413 | 4.9674 |  |  |  |  |
|  | 57913529 | 7.6971 | 6.5089 | 6.2436 |  |  |  |  |
|  | 57913662 | **47.0915** | **56.5374** | **53.7894** |  |  |  |  |
|  |  |  |  |  |  |  |  |  |
| 6-1 |  | RL20Na_50 | SDW20Na_50 | SDW20Na_200 | SFW20Na_50 | RDW20Na_50 |  | Sobic.006G064700  Chr06:42466729..42468514 reverse  RESPONSE REGULATORY DOMAIN-CONTAINING PROTEIN |
|  | 42464986 | 6.1320 | 6.0466 | 5.0326 | 7.2429 | 6.2518 |  |  |
|  | 42465246 | 5.7583 | 5.4674 | 5.1413 | 6.8006 | 5.6885 |  |  |
|  | 42465292 | 5.7583 | 5.4674 | 5.1413 | 6.8006 | 5.6885 |  |  |
|  | 42466875 | 3.1782 | 5.1512 | 4.8470 | 5.3512 | 4.7213 |  |  |
|  | 42467288 | 3.1652 | 5.1421 | 4.8489 | 5.3394 | 4.7408 |  |  |
|  | 42468175 | 3.0035 | 5.0302 | 4.7597 | 5.2419 | 4.5647 |  |  |
|  |  |  |  |  |  |  |  |  |
| 6-2 |  | RL19 | RL19Na_50 |  |  |  |  | Sobic.006G218850  Chr06:56601273..56601965 forward  unknown---sorghum-specific |
|  | 56596246 | 6.6813 | 5.3426 |  |  |  |  |  |
|  | 56596264 | 6.8709 | 5.6279 |  |  |  |  |  |
|  | 56600775 | 5.5205 | 5.0542 |  |  |  |  |  |
|  | 56611526 | 4.8645 | 5.2700 |  |  |  |  |  |
|  |  |  |  |  |  |  |  |  |
| 7-1 |  | RDW_STC_20Na_200 | SL_STC_20Na_200 | SFW_STC_20Na_50 | RL_STC_20Na_50 | RL_STC_20Na_200 |  | Sobic.007G050700  Chr07:5170031..5172247 reverse  unknown---sorghum-specific |
|  | 5169577 | 8.0041 | 7.8841 | 5.0039 | 5.0173 | **10.8669** |  |  |
|  | 5169584 | 8.0041 | 7.8841 | 5.0039 | 5.0173 | **10.8669** |  |  |
|  | 5169588 | 5.7465 | 7.3335 | 3.5674 | 4.4470 | **8.2821** |  |  |
|  |  |  |  |  |  |  |  |  |
| 7-2 |  | RDW_STC_20Na_200 | SL_STC_20Na_200 | SFW_STC_20Na_50 | SFW_STC_20Na_200 | RL_STC_20Na_50 | RL_STC_20Na_200 | Sobic.007G059400  Chr07:6213183..6217885 forward  Anthocyanin 5-aromatic acyltransferase  Sobic.007G059500  Chr07:6219129..6220244 reverse  Heading date 5- TF Y SUBUNIT B-2 |
|  | 6215246 | 5.7277 | 5.9140 | 7.0201 | **12.4984** | 4.5132 | 5.8977 |  |
|  | 6215646 | 7.3784 | 6.1786 | 7.5553 | 8.0532 | 7.2299 | **8.4625** |  |
|  | 6216018 | **9.6697** | **9.8635** | 4.4128 | 7.7289 | 6.1103 | **10.4470** |  |
|  | 6216025 | **9.5131** | **9.4805** | 4.5068 | 7.5377 | 6.0356 | **10.2824** |  |
|  | 6216438 | 5.3440 | 5.3443 | 6.4265 | 11.0186 | 4.2448 | 5.4841 |  |
|  |  |  |  |  |  |  |  |  |
| 8-1 |  | SFW_STC_19Na_50 | SFW_STC_19Na_150 | SFW_STC_19Na_200 |  |  |  |  |
|  | 32394666 | **44.6116** | **35.8466** | **45.2951** |  |  |  |  |
|  | 32394669 | **45.1763** | **37.3162** | **46.5496** |  |  |  |  |
|  | 32394671 | **45.1949** | **35.8323** | **44.9991** |  |  |  |  |
|  | 32394753 | **45.7916** | **36.3685** | **47.4643** |  |  |  |  |
|  | 32394769 | **45.9294** | **36.6161** | **48.0865** |  |  |  |  |
|  | 32394773 | **46.3031** | **37.0203** | **48.4608** |  |  |  |  |
|  | 32394789 | **46.2664** | **37.1039** | **48.1397** |  |  |  |  |
|  | 32394791 | **45.9590** | **36.8878** | **48.0214** |  |  |  |  |
|  | 32394793 | **46.9243** | **37.3560** | **48.5342** |  |  |  |  |
|  | 32394797 | **46.9675** | **37.4761** | **48.4366** |  |  |  |  |
|  | 32394808 | **45.9109** | **36.8619** | **48.1545** |  |  |  |  |
|  | 32394835 | **46.7780** | **37.1346** | **48.3141** |  |  |  |  |
|  | 32394916 | **46.0912** | **36.5648** | **47.8423** |  |  |  |  |
|  | 32394922 | **46.2954** | **36.7855** | **47.8919** |  |  |  |  |
|  | 32394951 | **46.9770** | **37.5526** | **48.6724** |  |  |  |  |
|  |  |  |  |  |  |  |  |  |
| 9-1 |  | RDW_STC_19Na_50 | RDW_STC_19Na_100 | RDW_STC_19Na_150 |  |  |  | Sobic.009G013000  Chr09:1131536..1136798 reverse  NACHT domain nucleoside triphosphatase  Sobic.009G013100  Chr09:1153321..1158646 reverse  NACHT nucleoside triphosphatase |
|  | 1141641 | 7.8889 | **8.3063** | **8.2811** |  |  |  |  |
|  | 1141830 | 7.4524 | 7.6408 | 7.4958 |  |  |  |  |
|  | 1144446 | **9.7868** | **10.5587** | **10.3842** |  |  |  |  |
|  | 1144535 | **10.6472** | **11.4521** | **10.4439** |  |  |  |  |
|  | 1144659 | 8.0594 | 7.2349 | 7.4808 |  |  |  |  |
|  | 1144675 | **14.2947** | **14.9127** | **13.3349** |  |  |  |  |
|  | 1144851 | **16.7367** | **16.8792** | **15.1042** |  |  |  |  |
|  | 1144894 | **8.7687** | **9.0048** | 7.3654 |  |  |  |  |
|  | 1145639 | **9.2637** | **10.8121** | **9.6345** |  |  |  |  |
|  |  |  |  |  |  |  |  |  |
| 9-2 |  | RFW19Na_100 | RL19 | RL19Na_50 | RL19Na_100 | RL19Na_150 |  | Sobic.009G015166  Chr09:1344483..1350632 forward  DUF247-Exhibits root specific expression  Sobic.009G015200  Chr09:1373052..1390135 reverse  unknown---sorghum-specific; exhibits root specific expression |
|  | 1342789 | 5.1402 | 5.4295 | 5.4752 | 5.9370 | 6.3164 |  |  |
|  | 1345695 | 5.1347 | 5.5003 | 5.4776 | 5.9436 | 6.2615 |  |  |
|  | 1347737 | 5.1402 | 5.4295 | 5.4752 | 5.9370 | 6.3164 |  |  |
|  | 1348108 | 5.1402 | 5.4295 | 5.4752 | 5.9370 | 6.3164 |  |  |
|  | 1349130 | 5.1620 | 5.4626 | 5.4974 | 5.9631 | 6.3188 |  |  |
|  | 1349692 | 5.1402 | 5.4295 | 5.4752 | 5.9370 | 6.3164 |  |  |
|  | 1364063 | 5.1620 | 5.4626 | 5.4974 | 5.9631 | 6.3188 |  |  |
|  | 1365517 | 5.0392 | 5.2855 | 5.3452 | 5.8234 | 6.2290 |  |  |
|  | 1369195 | 5.6171 | 5.6731 | 5.6738 | 6.3785 | 7.0757 |  |  |
|  | 1372555 | 5.5690 | 5.4853 | 5.1859 | 5.9419 | 5.7424 |  |  |
|  | 1378482 | 4.9139 | 4.9767 | 5.2621 | 6.0022 | 6.2096 |  |  |
|  | 1381710 | 6.3468 | 6.3367 | 5.7640 | 4.9731 | 5.7339 |  |  |
|  |  |  |  |  |  |  |  |  |
| 9-3 |  | SL_STC_19Na_150 | SL_STC_19Na_200 | SDW_STC_19Na_200 |  |  |  | Sobic.009G211200  Chr09:55730348..55734639 forward  tRNA(His) guanylyltransferase |
|  | 55732650 | 7.9613 | **9.3203** | **8.2641** |  |  |  |  |
|  | 55732654 | 6.5774 | **8.2847** | 6.7712 |  |  |  |  |
|  | 55732682 | 4.5799 | 5.3492 | 4.0325 |  |  |  |  |
|  |  |  |  |  |  |  |  |  |
| 9-4 |  | SFW_STC_19Na_50 | SFW_STC_19Na_150 | SFW_STC_19Na_200 |  |  |  | Sobic.009G256800  Chr09:59087688..59090509 reverse  unknown---grass-specific |
|  | 59082720 | **9.3244** | **9.5598** | **11.6120** |  |  |  |  |
|  | 59083139 | **11.0261** | **8.8094** | **9.6224** |  |  |  |  |
|  | 59084158 | **28.6661** | **23.6657** | **34.5696** |  |  |  |  |
|  | 59084167 | **15.6372** | **14.5886** | **17.7144** |  |  |  |  |
|  | 59084276 | **10.5617** | **8.3380** | **8.9468** |  |  |  |  |
|  | 59086808 | **12.0051** | **10.4605** | **10.6840** |  |  |  |  |
|  | 59086880 | **10.0882** | **10.4245** | **12.2185** |  |  |  |  |
|  | 59086913 | **12.9835** | **12.3071** | **16.5057** |  |  |  |  |
|  |  |  |  |  |  |  |  |  |
| 10-1 |  | RDW_STC_19Na_50 | RDW_STC_19Na_100 | RDW_STC_19Na_150 |  |  |  | Sobic.010G025850  Chr10:2094286..2114607 reverse  Myeloid leukemia factor 1-interacting protein |
|  | 2102398 | **19.4883** | **17.7749** | **17.8276** |  |  |  |  |
|  | 2102420 | **11.4629** | **10.6970** | **11.1729** |  |  |  |  |
|  | 2102443 | **11.4629** | **10.6970** | **11.1729** |  |  |  |  |
|  | 2102487 | **19.4120** | **17.8671** | **17.6900** |  |  |  |  |
|  | 2102597 | **10.4861** | **9.9241** | **10.5699** |  |  |  |  |
|  | 2102624 | **10.7385** | **10.3013** | **10.7701** |  |  |  |  |
|  |  |  |  |  |  |  |  |  |
| 10-2 |  | SFW20Na_50 | SDW20Na_50 | SDW20Na_200 |  |  |  | Sobic.010G162500  Chr10:47975240..47980077 reverse  CO dehydrogenase flavoprotein-like, FAD-binding |
|  | 47969370 | 5.7901 | 5.8324 | 5.4064 |  |  |  |  |
|  | 47969666 | 5.7877 | 5.8349 | 5.4214 |  |  |  |  |
|  | 47971983 | 5.7579 | 5.8099 | 5.4192 |  |  |  |  |
|  | 47972708 | 5.7877 | 5.8349 | 5.4214 |  |  |  |  |
|  | 47973884 | 5.7657 | 5.8049 | 5.4088 |  |  |  |  |
|  | 47975937 | 5.7702 | 5.8056 | 5.4282 |  |  |  |  |
|  | 47976462 | 6.0644 | 6.1601 | 5.7228 |  |  |  |  |
|  | 47977326 | 6.7709 | 6.9531 | 6.6608 |  |  |  |  |
|  |  |  |  |  |  |  |  |  |
| 10-3 |  | RDW_STC_19Na_50 | RDW_STC_19Na_100 | RDW_STC_19Na_150 |  |  |  | Sobic.010G198500  Chr10:54156867..54157585 forward  glutamine dumper 4  Sobic.010G198600  Chr10:54174417..54175293 reverse  unknown---not sorghum-specific |
|  | 54163054 | 5.6159 | 5.7686 | 5.6063 |  |  |  |  |
|  | 54164983 | **12.3078** | **12.4942** | **11.6923** |  |  |  |  |
|  | 54165760 | **12.9474** | **13.6254** | **13.0354** |  |  |  |  |
|  | 54167592 | **17.2769** | **17.3508** | **16.4552** |  |  |  |  |
|  | 54167866 | **12.3078** | **12.4942** | **11.6923** |  |  |  |  |
|  | 54170741 | **12.3078** | **12.4942** | **11.6923** |  |  |  |  |
|  | 54170988 | **12.1975** | **12.2741** | **11.5071** |  |  |  |  |
|  |  |  |  |  |  |  |  |  |
| 10-4 |  | RL20Na_200 | RFW20Na_200 | SDW20Na_200 |  |  |  | Sobic.010G234400  Chr10:57710675..57712367 reverse  O-methyltransferase ZRP4  root-specific expression |
|  | 57703966 | 5.3108 | 7.7432 | 5.1228 |  |  |  |  |
|  | 57706877 | 5.3316 | 7.7970 | 5.1187 |  |  |  |  |
|  | 57709212 | 5.1201 | 7.4280 | 5.1930 |  |  |  |  |
|  | 57709229 | 5.1863 | 7.3528 | 5.0367 |  |  |  |  |
|  | 57710773 | 5.0140 | 7.2024 | 4.8475 |  |  |  |  |

* The first part of each locus denotes chromosome number. -log(*P*) values in bold are above the threshold.
